# Diagnostic Assays for Avian Influenza Virus Surveillance and Monitoring in Poultry

**DOI:** 10.3390/v17020228

**Published:** 2025-02-06

**Authors:** Shahan Azeem, Kyoung-Jin Yoon

**Affiliations:** 1 Department of Veterinary Microbiology and Preventive Medicine, Iowa State University, Ames, IA 50011, USA; 2 Institute of Microbiology, Faculty of Veterinary Science, University of Veterinary and Animal Sciences, Lahore 54000, Pakistan; 3 Department of Veterinary Diagnostic and Production Animal Medicine, Iowa State University, Ames, IA 50011, USA

**Keywords:** avian influenza virus, surveillance, domestic birds, detection tools and tests

## Abstract

Diagnostic testing plays a key role in a surveillance program as diagnostic testing aims to accurately determine the infection or disease status of an individual animal. Diagnostic assays for AIV can be categorized into four broad types: tests for detecting the virus, its antigen, its genomic material, and antibodies to the virus. Virus characterization almost always follows virus detection. The present article surveys the current literature on the goals, principles, test performance, advantages, and disadvantages of these diagnostic assays. Virus isolation can be achieved using embryonating eggs or cell cultures in a lab setting. Virus antigens can be detected by antigen-capturing immunoassays or tissue immunoassays. Viral RNA can be detected by PCR-based assays (gel-based reverse transcription–polymerase chain reaction (RT-PCR), or probe or SYBR^®^ Green-based real-time RT-PCR), loop-mediated isothermal amplification, *in situ* hybridization, and nucleic acid sequence-based amplification. Antibodies to AIV can be detected by ELISA, agar gel immunodiffusion, hemagglutination inhibition, and microneutralization. Avian influenza virus can be characterized by hemagglutination inhibition, neuraminidase inhibition, sequencing (dideoxynucleotide chain-termination sequencing, next-generation sequencing), genetic sequence-based pathotype prediction, and pathogenicity testing. Novel and variant AIVs can be recognized by DNA microarrays, electron microscopy, mass spectroscopy, and Biological Microelectromechanical Systems. A variety of diagnostic tests are employed in AIV surveillance and monitoring. The choice of their use depends on the goal of testing (fit for purpose), the time of testing during the disease, the assay target, the sample matrix, assay performance, and the advantages and disadvantages of the assay. The article concludes with authors’ perspective of the use of diagnostic assays in the surveillance and monitoring of AIV in poultry.

## 1. The Virus

The avian influenza virus belongs to the genus *Alphainfluenzavirus* of the family *Orthomyxoviridae* [1,2]. The only species in this genus is *Influenza A virus*. Owing to their significance for human and animal health, influenza A viruses (IAVs) are the most studied influenza viruses. Avian influenza virus (AIV), which is the topic of this review, is also an influenza A virus and is also referred to as avian IAV. Influenza virus isolates are named after their genera, species of origin (except human isolates), location of positive animal or virus isolation made, a unique ID for the isolate, the year of isolation (e.g., A/Avian/US/xyz/2018) and, in case of influenza A virus, the subtype (e.g., H5N2).

The IAVs have eight segments of negative-sense, single-stranded RNA. While each segment codes for at least one structural or non-structural viral protein, a couple of RNA segments encode for more than one protein. The lack of proofreading by viral polymerase during replication results in the accumulation of point mutations that lead to minor changes in its antigenicity (antigenic drift). The segmented genome of IAVs paves the way for genetic reassortment between virus strains, which can occur when a host cell is co-infected with two or more IAVs—a mixed infection. Genome segments can be swapped between IAVs co-infecting the host cell (antigenic shift), often creating new IAV subtypes or variants that may have the ability to cross the species barrier.

The details about the IAV genome and proteins can be found elsewhere [3,4]. The surface protein hemagglutinin (HA) plays a critical role in the viral attachment to host cell receptors. In contrast, neuraminidase (NA), another surface protein, mediates the release of progeny virions from the host cells. While AIVs preferentially bind to the host cell’s sialic acids in α2,3 linkage (SAα2,3-galactose) with the penultimate galactose, the mammalian IAVs preferentially bind to those in α2,6 linkage (SAα2,6-galactose) [5,6]. Since attachment to receptors is crucial for initiating infection and replication, this binding specificity partially determines the host range of IAVs. Apart from the HA’s role in attachment to the cellular receptors, hence mediating virus entry in the host cell, its cleavage is critical for IAV infection, leading to productive replication [7]. The HA and NA proteins also form the basis for subtyping IAVs. IAVs can be further classified into a combination of 18 HA and 11 NA subtypes based on antigenicity and sequence similarity [8].

The distinct disease caused by AIV has been known since 1878 [9]. The 1961 outbreak in South African common terns was the first event to suggest the potential role of migratory wild birds in spreading AIVs to poultry [10]. The disease by AIV has been reported in all continents [11]. The AIVs are classified into either highly pathogenic AIV (HPAIV) or low-pathogenic AIV (LPAIV) relative to their pathogenicity in gallinaceous poultry. The LPAIVs of H5 and H7 subtypes can evolve to HPAIV due to genetic mutations of the HA gene (also known as Segment 4) [12]. The HA protein of HPAIV has several basic amino acids at the cleavage site. The HA of HPAIV can be cleaved by furin proteases, widely available throughout the host’s body, leading to systemic infection [13]. Conversely, the HA protein of LPAIV contains a single arginine or lysine at the cleavage site, restricting its cleavage by trypsin-like proteases, which are available only in the epithelial cells of respiratory and gastrointestinal tracts, leading to LPAIV infection limited to those tissues [7]. Mutation in the cleavage site, adding more basic amino acids, can convert LPAIV to HPAIV [7].

## 2. AIV Surveillance and Monitoring

### Terminology

Surveillance and monitoring are complementary concepts aimed at detecting a disease in a population and detecting changes in the patterns of a disease over time, respectively. Both concepts are used interchangeably in the literature. In this literature review, however, the concepts are used following the World Organisation of Animal Health (WOAH; formerly OIE, Office International des Epizooties) *Terrestrial Animal Health Code*, Thursfield et al. (2018), and Sergeant and Perkins (2015) [14,15,16]. Surveillance encompasses the “systematic, ongoing collection, collation, and analysis of information related to animal health, its timely analysis, interpretation, and dissemination to people requiring it, including those responsible for control measures so that that action can be taken” [14,15]. Surveillance can be differentiated into passive and active. Passive surveillance refers to the ordinary surveillance routinely performed by noticing clinical signs or bird’s mortality by farm workers that are reported to a veterinarian. In other words, the data collection during passive surveillance results from more general animal disease investigation activities. Because of its ongoing nature, it has proven very useful in detecting emerging diseases early and general trends of diseases over time in a population [16]. The major limitation of passive surveillance involves the high risk of underreporting diseases. On the other hand, active surveillance is proactive, involving frequent and intensive efforts to establish the presence or absence of a specific disease in a population [17]. This surveillance mode provides a more comprehensive estimate of disease occurrence and triggers specific intervention measures when the disease occurs; hence it incurs a higher immediate cost in terms of labor and time than its passive counterpart. The extra cost of active surveillance limits its use for short periods of time and specific purposes [18]. However, in the longer run, active surveillance offers potential savings in averting massive economic losses from high mortality due to a disease spreading to a flock, mass vaccinations, and access to markets that require proof of a disease-free status for exports and imports [17].

In practice, the distinction between surveillance and monitoring often becomes blurred. However, the differentiation relates more to the aims of an activity rather than the methods used for its implementation. Monitoring encompasses “intermittent performance and analysis of routine measurements and observations, aimed at detecting changes in the environment or health status—disease, productivity and possibly other characteristics related to them—of a population” [14,15]. Monitoring does not involve efforts to prevent or control disease. On the other hand, surveillance encompasses routine testing and supervision and a plan for prevention and control of the diseases being monitored when they occur; thus, its scope is broader. In the field conditions, monitoring is performed after surveillance efforts have suggested the presence or spread of disease. Most of the methods used for monitoring and surveillance can be used interchangeably.

## 3. Diagnostic Assays for AIV Surveillance and Monitoring

Many factors influence the success of a surveillance program. Among them, the choice of the right diagnostic test is a key factor. The goal of diagnostic testing is to accurately determine the infection or disease status of an individual animal or in a population. While a poultry flock’s disease status can be determined by observing clinical signs or changes in production parameters, the disease confirmation should be made by sensitive and specific diagnostic assays on a population subset. For AIV surveillance, types of assays comprise four broad categories: assays for detecting the virus, its antigen, its genomic material, and antibodies against the virus. Virus characterization almost always follows virus detection. The following section discusses these assays. Notes on miscellaneous assays used to detect variant and novel AIVs and the application of diagnostic assays for AIV surveillance and monitoring follow.

### 3.1. Assay to Detect Viable Virus

#### Virus Isolation

Virus isolation (VI) is the standard diagnostic method for AIV to confirm the presence of viable (i.e., infectious) viruses in specimens collected from suspect birds. Spackman and Killian (2020) have detailed protocols for attempting AIV VI [19]. In brief, the procedure for VI in embryonated eggs is as follows. Specific-pathogen-free (SPF) 9- to 11-day-old embryonated chicken eggs (ECE) are inoculated via the chorioallantoic sac route with the test material (i.e., specimen). Oropharyngeal–cloacal (OPC) and tracheal swabs from suspected birds are commonly used for VI. For LPAIVs, lungs, intestinal contents, and mixed viscera homogenates can be used [20,21]. For HPAIVs, additional tissues, such as the brain, can also be used [21].

The inoculated eggs are monitored daily for embryonic death for at least 5 days post-inoculation. The amnio-allantoic fluid (AAF) is harvested soon after embryo death is recorded or at the end of a 5-day incubation period—whichever comes first. The AAF is tested by a hemagglutination test for the presence of an agent agglutinating chicken red blood cells (RBCs). A reverse transcription–polymerase chain reaction (RT-PCR) assay should be then used to confirm the presence of IAV because paramyxoviruses, such as avian paramyxovirus type 1 (also known as Newcastle disease virus), can also agglutinate chicken RBCs.

A study has demonstrated that embryonated chicken, turkey, or duck eggs can be used interchangeably to isolate LPAIVs from wild birds [22]. Additionally, 28-day-old ostrich embryonated eggs, besides embryonated eggs of the avian species mentioned above, have also been used [23].

Virus isolation in ECE is a highly sensitive method for detecting AIVs in clinical samples but requires confirmatory testing as avian pathogens other than AIV can inadvertently grow in ECE. Some downsides of VI in ECE are the following: (1) VI in ECE is time-consuming (7–14 days), laborious, expensive, and requires advanced technical skills. It cannot be easily scaled up unless the necessary infrastructure (e.g., egg supplier, technical staff, and incubator) is in place beforehand; and (2) given that the virus can be grown to a high titer in eggs, there are increased chances of cross-contamination to negative samples or inadvertent exposure of the laboratory personnel [24]. Despite these drawbacks, ECE is a more efficient system for isolating LPAIVs of wild bird origin than cell culture-based systems [22,25]. Some cell lines, such as Madin–Darby Canine Kidney (MDCK), African green monkey kidney (Vero), fibroblastic human embryo lung (MRC-5), mink lung epithelial cells (Mv1Lu), and human colorectal adenocarcinoma cells (Caco-2), can also be used to cultivate avian and mammalian IAVs [26,27,28,29,30]. MDCK cells are preferable for isolating swine lineage IAVs of turkeys [19]. Virus isolation remains the only laboratory method to obtain a viable virus for downstream testing, such as virus characterization and pathogenicity studies. Therefore, VI has been a critical part of AIV surveillance.

### 3.2. Assays to Detect Viral Antigen

#### 3.2.1. Antigen-Capturing Immunoassays

Antigen-capturing immunoassays (ACIAs) can be employed in a lateral flow or micro-well format [24]. In a lateral flow format, a monoclonal antibody (mAb) directed against a highly conserved epitope on nucleoprotein (NP) or matrix (M) protein is used to capture AIV in the test sample on a filter paper strip or membrane. Monoclonal antibodies (mAbs) are preferred because of their high specificity, even though polyclonal antibodies specific for NP or M protein can also be used as capturing antibodies. A second mAb chemically bound to either colored latex beads or colloidal gold particles is used for detection [24,31]. An assay kit using a combined gold nanoparticle mAb conjugate targeting both NP and M protein can be more sensitive than ones targeting a single protein, either NP or M protein [32].

The lateral flow ACIA (also known as lateral flow device) is rapid, giving results in approximately 15 min, and is portable; hence, such a test kit can be used as a pen-side testing tool [i.e., point-of-care (POC) test] for AIV [24,33]. The test is often referred to as a rapid antigen test (RAT). Under the National Poultry Improvement Plan (NPIP), the United States Department of Agriculture (USDA) has approved using RAT kits for AIV on clinically ill or dead chickens and turkeys in a flock on-site [34]. Owing to their poor sensitivity, ACIA-negative birds must be further tested with a more sensitive assay such as real-time RT-PCR (rRT-PCR) or VI. Samples from positive flocks, as determined by PCR assay or VI, are then further tested by a federal reference laboratory for verification. The final confirmation is based on additional sampling and testing with suitable confirmatory assays [34].

Although poorly sensitive, ACIA is highly specific for detecting AIVs. Another useful feature of ACIA is its commercial availability. However, developing a subtype-specific antigen detection test is still a challenge. Since the HA and NA proteins of AIV, which form the basis of its subtyping, undergo continuous unpredictable mutations, periodic updates of antibodies to these proteins in a kit are required, which is a significant impediment to developing a subtype-specific antigen detection kit [35].

The analytical sensitivity of ACIA kits is generally low, requiring the presence of a virus at approximately ≥10^4^ mean embryo lethal dose (ELD_50_)/mL or median embryo infectious dose (EID_50_)/mL in the sample being tested [36]. Therefore, ACIA may perform well for clinically ill and heavy shedders (e.g., HPAIV-infected birds) but may not detect subclinically infected poultry that tends to shed a low level of virus, thus leading to false negatives. This limits the use of ACIA for routine AIV surveillance or as a screening tool [36]. Another limitation of ACIA is that most of the kits are designed to test tracheal or oropharyngeal swabs, so fecal samples may not work well on these kits or cause a false positive result.

#### 3.2.2. Immunohistochemistry

An immunohistochemistry (IHC) method can be used to detect a variety of AIV antigens in cells of a tissue section. Briefly, antibodies labeled with a dye or an enzyme and specific to antigen epitopes are used to visualize the virus using an enzyme chromogen reaction. Photos of intranuclear and intracytoplasmic immunohistochemical staining for avian influenza virus (H5N1) nucleoprotein in cerebral neurons and pancreas of an experimentally infected duck can be found in the following publication [37]. The details of the technique are available elsewhere [38,39,40]. One advantage of IHC is that it enables the direct morphological localization of AIV antigens in tissues from infected animals. Another advantage is the use of formalin-fixed, paraffin-embedded tissue for IHC, which reduces the risk of operator exposure to zoonotic AIVs. In addition, its high sensitivity allows the identification of AIVs before morphological changes in tissues appear. Besides, IHC offers the possibility of retrospective diagnosis and in-depth study of the disease [38].

### 3.3. Assays to Detect Viral RNA

#### 3.3.1. Reverse Transcription–Polymerase Chain Reaction

PCR-based assays are commonly used to detect viral genomic material in various sample matrices. Reverse transcription–polymerase chain reaction (RT-PCR) assay is a rapid test for AIV screening or subtyping of OPC or tracheal swabs and tissues from wild birds or poultry [41]. PCR-based assays can be run in conventional (i.e., PCR amplification followed by gel electrophoresis to visualize PCR products) or real-time (i.e., PCR amplification and visualization of PCR products at the same time) formats. The PCRs are commonly followed by sequencing to confirm the presence of AIV-specific amplicons and to obtain the genetic information of segments.

Real-time RT-PCR is generally quicker and more sensitive than conventional RT-PCR. The use of additional oligonucleotides known as probes provides an additional layer of specificity to the assay since primer non-specific binding does not result in a positive signal unless the probe also binds to the amplified target. Real-time RT-PCR can be deployable with a specialized instrument and ready-to-use reagents for pen-side testing when needed. Several rRT-PCR platforms are available, as described below, depending on the chemistry used to detect PCR products or fluorochrome types.

##### Fluorescent Reporter Probe Methods

TaqMan^®^ rRT-PCR is based on hydrolysis probe chemistry [41]. In brief, in addition to specific primers, a probe with an oligonucleotide sequence complementary to the target genomic sequence is added to the PCR reaction. The probe has a fluorogenic reporter dye attached to a quencher dye. When the quencher dye is in close proximity to the reporter dye, it absorbs any light emitted by the reporter dye. The probe anneals to the target during PCR amplification. The *Taq* polymerase, owing to its exonuclease activity, cleaves off the quencher from the reporter dye, thus allowing it to emit its fluorescence when it encounters the light. The fluorescence can be recorded by a fluorimeter [41,42]. Different reporter dyes emit fluorescence at different wavelengths, which allows multiplexing to detect multiple targets simultaneously [43].

Another probe-based rRT-PCR uses a probe known as a molecular beacon (MB). Molecular beacon probes are 15–25 nucleotide long and flanked by two short complementary stem sequences, forming a hairpin-like, stem–loop structure that fluoresces when hybridizing to the RNA or DNA target. The stem–loop structure brings the quencher and fluorophore, located at opposite ends of the MB, into close proximity, effectively quenching the fluorescence. However, upon binding to the target nucleic acid, a conformational change in the probe distances the quencher from the fluorophore, allowing the latter to fluoresce [44]. Probes with different colored fluorophores, which target different sequences, can be used in a multiplex reaction to detect multiple targets simultaneously [45].

Molecular beacon probes have a few advantages over TaqMan^®^ probes: low fluorescence background, efficient fluorescence quenching by proximal quencher, and a higher amount of resonance energy transfer [46]. MB probes are not degraded during PCR amplification; hence, they are recyclable [47]. One disadvantage of the MB probe is its low sensitivity. This is because it must compete with the complementary strand, and only a portion can bind to its target. Kong et al. (2002) developed a modified MB with the combined properties of TaqMan^®^ that yielded higher sensitivity compared to conventional MBs [48]. Interestingly, when Wang et al. (2005) compared the sensitivity of TaqMan^®^ PCR and MB PCR, the authors found MB probes to be more sensitive than TaqMan^®^ probes. The high-fluorescence background of the TaqMan^®^ probe was speculated to be responsible for its low sensitivity [46].

##### SYBR Green Reporter Method

A SYBR^®^ Green-based rRT-PCR employs SYBR Green dye’s intercalating properties, which inserts itself in double-stranded, amplified DNA. It exhibits a stronger interaction with double-stranded DNA when compared to single-stranded DNA or RNA [42]. Hence, DNA amplification can be detected by a fluorescence signal from SYBR Green instead of using a probe. While SYBR Green-based PCRs are generally run as singleplex, multiplexing is also possible by running together for targets with different melting curves [49].

The simple chemistry of SYBR Green-based rRT-PCR makes it relatively easy to develop and perform, and it is significantly inexpensive compared to TaqMan^®^ or MB-based rRT-PCRs [50]. Yet, there are some limitations to SYBR Green-based PCR assays. Since the dye can be inserted in any amplified double-stranded DNA, the amplification signal can be produced if primers bind to a closely related non-target sequence. Because primer dimers’ formation can generate the fluorescent signal from SYBR Green, a melting curve analysis must be performed to differentiate the amplified signals of the target’s sequence from non-target sequences [51]. The sensitivity of SYBR Green-based rRT-PCR is relatively low compared to that of TaqMan^®^ or MB probe-based rRT-PCRs.

#### 3.3.2. Loop-Mediated Isothermal Amplification Method

Loop-mediated isothermal amplification (LAMP), as the name implies, involves amplifying a target sequence under isothermal conditions. The LAMP process uses a DNA polymerase and four to six primers, including two looping primers and two stripping primers [52,53]. Including a reverse transcriptase in LAMP (i.e., RT-LAMP) allows the assay to detect RNA. Specific LAMP assays have been developed for human H1 and H3, avian H5, H7, and H9 IAVs [54,55,56]. There has also been a report of simultaneous detection of human influenza A (H1 and H3) and B viruses using multiplex RT-LAMP assay [57].

LAMP assays can be performed optimally at ambient temperature. Additionally, LAMP assays do not require specialized equipment and reagents; therefore, the assay is useful for AIV surveillance in developing countries where infrastructural resources are scarce [42]. RT-LAMP assays in both conventional and real-time formats can be used to rapidly test clinical samples for AIV with high sensitivity [58,59]. These assays also have a great potential for pen-side testing. However, LAMP testing presents some limitations. First, designing 4–6 primers could be challenging. This is because, in addition to other requirements for primer design, LAMP primers necessitate selecting 6–8 distinct regions on the target gene [60]. Second, using multiple primers in the assay increases the chances of primer–primer interactions, potentially leading to false-positive results [61].

#### 3.3.3. Nucleic Acid Sequence-Based Amplification

Nucleic acid sequence-based amplification (NASBA) is an isothermal and enzymatic reaction especially designed for RNA targets as it is a transcription-based technique [62,63]. The whole amplification process is run at 41 °C. The maintenance of a constant temperature allows each step to proceed as soon as an amplification intermediate becomes available. The efficiency of NASBA reactions is based on multiple copies of RNA transcribed from a single DNA product [62].

**Figure 1:**
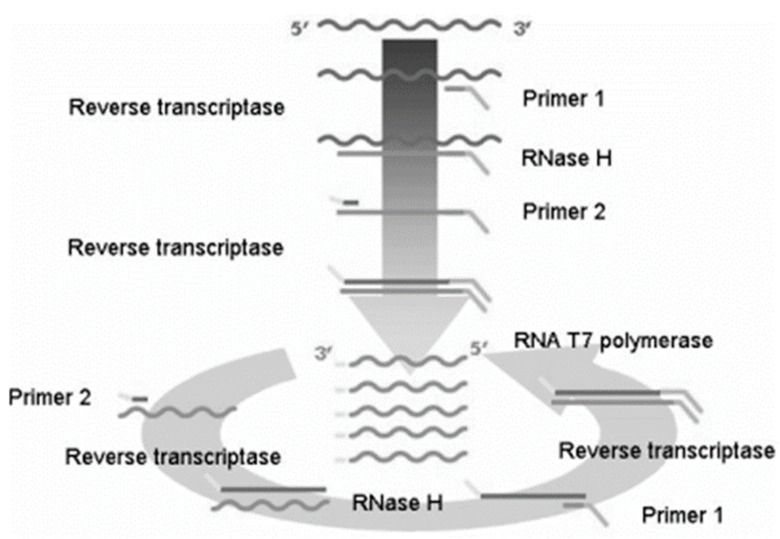
The Nucleic Acid Sequence-Based Amplification process (adopted from Rodríguez-Lázaro et al. 2006 with permission) [64]. The wavy lines show RNA, and the straight lines show DNA.

NASBA involves a reverse transcriptase (RT), T7 RNA polymerase, ribonuclease-H (RNase H), and two primers (P1 and P2). P1 binds to and synthesizes a complementary DNA (cDNA) from the viral RNA (vRNA) by reverse transcription, yielding a vRNA–cDNA hybrid. The vRNA strand of the hybrid is hydrolyzed by RNase H, leaving behind single-stranded cDNA. The P2 can then bind to the newly separated cDNA from the hybrid and elongate to yield a double-stranded DNA molecule (DNA duplex). The T7 RNA polymerase binds to the T7 promoter on Primer 1 and synthesizes multiple copies of RNA from the cDNA template. The amplified cDNA also contains a promoter for T7 RNA polymerase, which then binds to its promoter on the DNA, yielding many copies of RNA. The newly synthesized RNA molecules can then serve as templates for further rounds of reverse transcription, RNA degradation, and RNA synthesis leading to amplification of the target RNA sequence. A cyclic interaction between the two primers comprising the same events (as described above) takes place, except the primers bind in the reverse order, i.e., the P2 now binds to newly synthesized copies of RNA. The overall reaction results in approximately 10^9^-fold amplification in 1–2 h (Figure 1) [64].

Advantages of NASBA include its high precision without expensive equipment and higher sensitivity than RT-PCR [63]. Even though the NASBA assay is specific, rapid, and sensitive to detect H5 AIVs (both high and low pathogenic strains), its widespread use has been greatly limited because of the high cost of the commercial kits, the in-house preparation of the master mix, and several required optimization steps [42].

### 3.4. Assays to Detect AIV-Specific Antibodies

Poultry sera are often tested for antibodies specific to AIV to ascertain a commercial poultry flock’s immune status after vaccination in countries practicing AIV vaccination. AIV vaccination of poultry is not practiced in the USA. The presence of AIV antibodies is also indicative of past infection or the presence of passively transferred maternal antibodies. Besides molecular tests, antibody testing can be used to certify an area or premises is free of AIV, or during an AIV outbreak to determine the magnitude of the infected zone for quarantine purposes.

Antibodies can be detected using various serological methods, including serum-virus neutralization (SVN) test, enzyme-linked immunosorbent assay (ELISA), agar gel immunodiffusion (AGID) test, and hemagglutination inhibition (HI) test. Regarding screening commercial poultry for AIV, a regulatory agency would use an assay with high sensitivity and high throughput first, such as an ELISA. Positive samples are confirmed using an assay with high specificity, such as the AGID test, even though it is of low throughput moderate/low sensitivity. The confirmed positive samples are then evaluated by an HI test to determine the subtype of AIV to which birds were exposed. Serologic testing using the AGID or HI test is relatively inexpensive compared to ELISA.

In avian influenza-endemic countries, seroconversion or rising antibody titers to AIV, when paired sera are used, provide indirect evidence of exposure to the virus. Paired sera are two serum samples taken at least 2 weeks apart from the same bird or flock. The first is taken during the acute phase, and the second is taken during the convalescence phase. Either of the following two changes in the serologic profile is suggestive of infection: if there is a four-fold or greater increase in titer between the acute and convalescent sera or if there is a change of status from negative to positive between the acute and convalescent sera (i.e., seroconversion). Additionally, flock monitoring after vaccination involves ascertaining the development of a protective antibody titer.

#### 3.4.1. Serum-Virus Neutralization Test

Serum-virus neutralization test is an assay to detect AIV-specific antibodies that can neutralize the virus infection to permissive cells. It is commonly run in a microneutralization format. In this format, serum containing antibodies to AIV is serially diluted and mixed with a fixed amount of the virus. The antibody–virus mixtures are then incubated with cells susceptible to AIV. The virus neuralization is confirmed by the absence of cytopathic effects in the inoculated cells or histochemical staining by antibodies specific to the virus. Photos of AIV-induced CPE in MDCK cells can be found in the following publication [65]. The assay is relatively inexpensive as standard laboratory equipment can be used. However, the use of cell culture makes it time- and labor-intensive. A study comparing the microneutralization test and the HI test indicated that the former was more sensitive in detecting human antibodies to the H5N1 virus in infected individuals [66].

#### 3.4.2. Enzyme-Linked Immunosorbent Assay

The enzyme-linked immunosorbent assay is a rapid test for screening poultry sera for IAV antibodies. Various sample matrices such as a serum, plasma, and eluate of dried blood collected on a filter paper can be tested by ELISA [67,68,69]. ELISA can also test egg yolk, but the AGID test may work better with yolk samples. A commercially available NP-blocking ELISA detects IgG antibodies to IAV NP in samples from any species [70]. HA and NA subtype-specific antibody ELISAs have also been developed but are of limited commercial use due to rampant mutations in HA and NA proteins of AIV [71,72,73,74,75,76]. Compared to the AGID test, ELISA is superior in high throughput capability, built-in quality control, and semi-automated operation. Additionally, ELISA is reported to have higher sensitivity compared to AGID and HI tests for IAV antibodies [77,78].

Disadvantages of ELISA include its requirement of expensive equipment such as an ELISA plate reader. ELISA’s specificity is relatively low compared to that of the AGID test even though direct comparisons are difficult because the AGID test is specific for IgM and ELISA detects IgY; therefore, ELISA-positive samples require confirmation with a more specific test such as the AGID test [79]. Recently, Jensen et al. (2017) found an excellent correlation between a commercial competitive ELISA (ID Screen^®^, IDvet, 34,790 Grabels, France) and HI test for detecting anti-H5 antibodies in Danish zoo birds, suggesting that this ELISA can be used in screening birds for antibodies against H5 AIV [80]. Yet, it should be noted that a high degree of antigenic variations in HA protein among AIVs may lead to false negatives in these tests.

#### 3.4.3. Agar Gel Immunodiffusion Test

The agar gel immunodiffusion test is generally designed to detect antibodies to highly conserved AIV antigens, such as NP or the M protein, in infected poultry. Serum, plasma, or egg yolk can be tested for AIV antibody using the AGID test [81]. Egg yolks are convenient samples that can quickly be submitted to a diagnostic laboratory for testing, whereas serum collection is relatively time-consuming and laborious. The assay can detect circulating immunoglobulin M (IgM) in chicken and turkey blood within 5–7 days after infection [81]. Although the AGID test is easy to set up and can test multiple species, the test is not reliable for detecting antibodies in ducks and other wild birds because the immune response in these species varies, and antibody production to NP is not consistent [82,83].

The test is commonly set using a six-well pattern around a central well in an agar gel. AIV antigen is added to the central well, while reference (known positive and known negative sera) and test sera (generally 1:10 or 1:20 diluted) are added in the peripheral wells. A visible line of precipitation between the central and peripheral wells after 24 h of incubation indicates a positive test result. A photo of the immunodiffusion test pattern with AIV can be found in the following publication [84].

Using the AGID test, Trample et al. (2006) found that 20 weeks after experimental infection, 90% of serum samples from chickens inoculated with a H6N2 LPAIV isolate were positive, but only 43% of eggs from infected hens were positive [85]. A low-pathogenic AIV field outbreak also indicated that the antibody seems to persist longer in serum than in egg yolk [86]. This finding suggests that the AGID test on egg yolks performs best if testing is performed earlier during the outbreak, but its sensitivity decreases as the outbreak progresses. Taken together, these studies demonstrate that serum samples are a more reliable sample matrix for the AGID test than eggs when initial exposure to AIV is expected to be weeks or months earlier than the sample collection, even though eggs can be a convenient sample for testing.

The AGID test is used to screen poultry for AIV exposure, and it is the WOAH’s reference test for serologic diagnosis of AIV infection. This assay requires a few reagents and equipment; therefore, it is less expensive than ELISA [84]. The test has a high diagnostic specificity or low false-positive rate. Therefore, in several US states, LPAI serology programs for domestic poultry require testing all birds positive by commercial blocking ELISA with AGID test. The AGID test result is interpreted at the flock level and not at the individual level since the test result suggests a flock’s immunological status.

Disadvantages of the AGID test include its moderate sensitivity, which is lower than that of ELISA, and the subjectivity involved in interpreting results [77]. Since the AGID test is semi-quantitative, it does not allow determining the end-point antibody titer. Another drawback is that the test results are available after 24–48 h of setting up.

#### 3.4.4. Hemagglutination Inhibition Test

Avian influenza viruses have varying abilities to agglutinate red blood cells (RBCs) of various animal species (hemagglutination). Hemagglutination inhibition (HI) is based on the principle that antibodies to AIV (mainly HA protein) interfere with the hemagglutination activity of the virus. Since the detection of HI antibodies can be used to indicate the presence of neutralizing antibodies in the birds [87], vaccinated birds can be monitored for neutralizing antibodies to AIV using the HI test instead of the SVN test. Briefly, test serum from vaccinated birds is serially diluted two-fold in a 96-well U-bottom plate, followed by the addition of a uniform amount (four hemagglutination units) of the virus. After briefly mixing the plate contents by using a laboratory shaker or by manual agitation, the plates are incubated at room temperature for 30 min. Finally, 0.5–1.0% suspensions of washed chicken, turkey, or guinea pig RBCs are added. After a 30-min incubation period at room temperature, the results can be recorded for analysis. A clear button formation at the bottom of the well indicates HI, while hemagglutination is indicated by a mat formation of RBCs by the virus [88]. A photo of the hemagglutination inhibition test result can be found in the following publication [89].

The HI titer is recorded as the reciprocal of the highest serum dilution showing HI activity. HI titer is expressed as a geometric mean titer (GMT) and is generally reported on a group basis. WOAH has laid out vaccine efficacy requirements based on the GMT of vaccinated birds as determined by the HI test. According to these requirements, a GMT of ≥32 is associated with the prevention of mortality in poultry, and a GMT of ≥128 is associated with a reduction in challenge virus replication and shedding [20].

Here are some limitations of the HI test for quantitation. To achieve precise quantification, it is essential that the serum antibody and viral antigen are antigenically compatible. Any discrepancies between the serum antibody and viral antigen can reduce the accuracy of the results, besides increasing test result variability due to the lack of standardization of protocols among laboratories [88,90], highlighting the necessity for homologous AIV strains for quantification. As a result, HI results should be considered only qualitatively when evaluating an antibody with an unknown antigenic specificity, rendering the HI assay unsuitable for screening antibodies of unknown subtype or lineage [89]. These limitations make the HI test difficult to interpret, especially when applied to surveillance and monitoring. Because of the cost to purchase reference antisera against various HA subtypes, the HI test can be expensive to run and may not be a feasible option for field screening of commercial flocks. Besides cost, availability of the serum is another issue in some countries, as such reference sera may not be available.

The HI test is rapid, easier to perform, and requires less technical expertise than the SVN test. The HI test can be performed on a large number of samples simultaneously, and HI reagents and equipment are readily available in many laboratories. However, the HI test is less sensitive than the SVN test, especially for detecting low levels of antibodies. While an HI antibody can be a surrogate for an SVN antibody, titers determined by these two tests are not always in agreement [88]. The HI test may not be able to distinguish between antibodies that neutralize the virus and those that do not. The HI test may not detect all types of antibodies important for protection against avian influenza. Overall, the HI test can be a good screening tool for detecting antibodies against AIV, but it may not be sensitive or specific enough for all purposes. In contrast, the SVN test is more sensitive and specific, but it is also more complex and time-consuming. The HI test is also relatively inexpensive compared to the SVN test, supporting its use in resource-constrained countries. The choice of test depends on the specific needs of the laboratory.

### 3.5. Assays and Methods to Characterize AIVs

#### 3.5.1. Subtyping

The subtype of AIV infecting birds can be determined using the HI test with an antiserum raised against the known HA subtype (i.e., reference antiserum). For testing, OPC swabs from infected birds or allantoic fluid from embryonated chicken eggs in which VI has been attempted is diluted by a serial 2-fold dilution technique in a 96-well U-bottom plate to which an equal amount of reference antiserum is added. The rest of the procedure and interpretation of the results are the same as outlined above in 7.4.

While the HI test is commonly run using poultry sera containing polyclonal antibodies against AIV, these sera may exhibit some background reactivity, which can interfere with test interpretation. Besides, the composition of polyclonal antibodies can vary between batches, potentially affecting test results. Although mAbs suffer from poor HI activity [91], their use offers several advantages over polyclonal antibodies for AIV characterization, such as high specificity (i.e., low false positives) and consistent properties enabling reproducibility, besides ease of production [92]. HI tests using mAbs (in the form of a mixture) are simple to perform, can be completed quickly, and do not require hi-tech equipment [93]. Anti-HA mAbs could help in the subtyping of IAVs [94]. Zeng et al. (2004) assert that mAbs can be a useful tool for AIV antigenic analysis [95].

When applied to AIV surveillance, the HI test can be used for antigenic cartography studies. These studies calculate antigenic distances between influenza viruses or serum antibodies by quantifying raw HI data, hence determining antigenic variants [96,97].

The NA subtype of AIV in clinical specimens or allantoic fluids from embryonated chicken eggs can be determined by neuraminidase inhibition (NI) assay [98]. The assay mechanic is founded on the enzymatic activity of the NA glycoprotein of AIV, which cleaves off sialic acid (cellular receptor for AIV) from the virus, allowing the virus to be released. For testing, a virus material is incubated with the known NA-specific antibody, and an NA substrate, such as fetuin, is added. The mismatch between the antibody and NA of the virus in the test sample cannot stop NA from breaking fetuin into free sialic acid. Further steps include the addition of periodate and, subsequently, sodium arsenate. Periodate reacts with free sialic acid to form beta-formylpyruvic acid, which turns pink when sodium arsenate is added—indicating that the test sample is negative for the specific NA subtype containing virus (i.e., no NI) [98]. However, if the antibody is specific for NA of AIV present in the test sample, it blocks viral NA from breaking fetuin, preventing downstream reactions yielding no color change (positive reaction). A photo of the neuraminidase inhibition test results with IAV can be found in the following publication [98]. Recently, the enzyme-linked lectin assay has been utilized for the detection and quantification of neuraminidase-inhibiting antibodies [99,100]. The NI assay is generally performed in a reference laboratory because of the requirement for specific reagents to run the assay.

PCR-based assays can also be used to characterize IAVs. The ability of a PCR to simultaneously detect multiple targets (i.e., multiplexing or panel testing) is advantageous for AIV subtyping as multiple AIV subtypes can be present in a bird concurrently [101]. This multiplexing ability can greatly enhance testing efficiency and AIV surveillance capability. An MB-based sensor system for AIV subtyping has been reported [102]. The assay was able to subtype HA and NA of AIVs from a sample with unpurified PCR amplicons. Subtyping directly from PCR amplicons without purification is a step towards efficient surveillance testing with fast turnaround.

The dideoxynucleotide chain-termination sequencing method, commonly known as Sanger sequencing, is a highly reliable subtyping technique for extremely variable HA and NA genes of AIV. While the sequencing could be performed directly using OPC swabs, an RT-PCR assay is generally performed to amplify the desired gene segment. Once an amplicon is available, it can be sequenced using the amplification primers or other sequencing primers to obtain the entire fragment’s nucleotide sequence. Alternatively, a smaller fragment amplified by PCR can be sequenced first to determine the presumptive HA or NA subtype. The remaining sequence of HA or NA fragments can be obtained using subtype-specific amplification and sequencing primers [103,104]. Upon completion of the sequencing, the subtype can be determined by sequence alignment and comparison with AIV sequences with known HA or NA subtypes available in public databases, such as GenBank^®^ (NCBI—National Center for Biotechnology Information), EpiFlu™ (GISAID—Global Initiative on Sharing All Influenza Data), and IRD (Influenza Research Database). The Basic Local Alignment Search Tool (BLAST^®^, NCBI) is commonly used for that purpose.

Sanger sequencing is fast and cost-effective for a low number of targets (1–20 targets). Sanger sequencing depends on successful PCR amplification and post-PCR purification of the PCR products. It requires that a portion of the genome is known for which PCR and sequencing primers can be designed. Therefore, it cannot be used to sequence novel pathogens. This technology has a low limit of detection and scalability due to increased sample input requirements.

Next-generation sequencing (NGS) technologies can be utilized for the surveillance of AIVs. NGS can be used to study the genetic diversity of AIVs [105]. NGS technologies can be especially helpful in the identification of novel AIVs or subtypes [106,107,108]. The NGS utilizes parallel sequencing of multiple small DNA fragments to obtain the complete sequence. The amount of sequence data generated by NGS is far greater and faster than Sanger sequencing; hence, NGS is also commonly referred to as high-throughput sequencing. Next-generation sequencing can be performed using any available platforms, such as Roche 454^TM^, Applied Biosystems SOLiD^®^, Illumina (HiSeq^TM^, MiSeq^TM^), Ion Torrent™, PacBio^®^ RS system, Oxford Nanopore, and so on. The process starts with pathogen enrichment. The host sequences and the bar code sequences are removed from raw sequence data to obtain pathogen sequences. The obtained sequences are then aligned to find overlaps between the reads—contigs, leading to the reconstruction of the original sequence. Contigs can be made by *de novo* or reference-guided assembly. In *de novo* assembly, contigs are created from scratch without external data, whereas in reference-guided assembly, contigs are made after aligning each read with a reference genomic sequence. Once AIV genome contigs are obtained, BLAST^®^ can then be used to search for similar sequences. Greninger et al. (2010) combined and assembled 90% of sequence data of the H1N1pdm09 genome, without a reference sequence, from all 17 outbreaks. The authors asserted that NGS could be a powerful tool for discovering novel pathogens and, by virtue of the simultaneous availability of host gene expression and co-infection data, for studying host–pathogen and pathogen–microbiota interactions [109]. In another study, Lee et al. (2017) used both *de novo* and reference-guided genome assemblies to successfully construct the entire genome of H7N8 HPAIVs and LPAIVs that affected commercial turkey flocks in Indiana in 2016 [110].

Compared to Sanger sequencing, NGS enables higher sequencing depth or sensitivity. Because it is independent of any knowledge about the genome sequence of a pathogen being investigated, NGS can be used for the discovery of novel pathogens and their sequences. NGS produces more data for a DNA sample run and is high-throughput compared to Sanger sequencing. However, analysis of NGS data to draw meaningful conclusions follows a tiered approach and requires the knowledge of specialized software for bioinformatic analyses. NGS is less cost-effective and time-consuming for sequencing low numbers (1–20) of targets. Cost is a major limiting factor in using NGS technologies for routine clinical diagnosis. However, Greninger et al. (2010) contended that NGS could generate much more valuable data on the host microbiome and gene expression, as well as the whole-genome sequence of the influenza virus, at a cost comparable to conventional PCR [109]. Additionally, Okamatsu et. al. (2016) suggested that NGS could be used in the future to investigate outbreaks caused by potential novel pathogens, including AIVs. A drawback of NGS technologies is that—as is the case with other molecular tests—they cannot differentiate between viable and non-viable viruses [42].

The NGS technologies have myriad applications in the diagnosis and surveillance of AIVs. For example, NGS can detect and identify AIVs directly from clinical samples, bypassing the need for virus isolation. It can also detect the whole genome of multiple AIV strains simultaneously, including novel or emerging strains, providing information on its subtype, lineage, and mutations. This information is crucial for understanding the virus’s evolution, transmission, and potential for causing zoonosis. The NGS enables large-scale surveillance of AIV in poultry populations and wild birds. It can track the spread of the virus and identify potential outbreaks early. NGS data can be used to form intervention strategies, such as vaccination and culling programs. The NGS data can also be used to develop new diagnostic tests. 

#### 3.5.2. Pathotype Prediction Based on Proteolytic Cleavage Site of HA Protein

The nucleotide sequence of the HA proteolytic cleavage site (PCS) of H5 and H7 AIVs can be used to predict their pathogenicity in chickens. If the PCS of an AIV matches the PCS of previously characterized HPAIV, the virus is notifiable as highly pathogenic as per WOAH criteria [20].

Pathotype prediction based on molecular signature alone, without *in vivo* testing, could be problematic. The absence of a proteolytic cleavage site in the HA protein of an AIV does not rule out a mixture of highly and low pathogenic viruses in the test sample [20]. Furthermore, at least two H10N4 and H10N5 isolates are known to cause over 87% mortality in susceptible chickens and have an intravenous pathogenicity index (IVPI) of >1.2 when inoculated intravenously but lack the proteolytic cleavage site sequence [111]. Conversely, there are four AIVs with the HA0 cleavage site containing multiple basic amino acids, but when inoculated intravenously into 6-week-old chickens. They showed low pathogenicity with IVPI < 1.2 [112]. These observations highlight the necessity for running *in vivo* trials to confirm the pathogenicity of an AIV isolate.

#### 3.5.3. Assessment of Pathogenicity

Animal inoculation, also known as bioassay, is used extensively for AIV pathogenesis studies [113]. The WOAH’s guidelines for *in vivo* pathogenicity testing used to categorize H5 or H7 as LPAIV or HPAIV for chickens stipulate that AIV is termed as highly pathogenic if its intravenous inoculation results in intravenous pathogenicity index (IVPI) of >1.2 (up to 3) and low pathogenic if the IVPI value is between 0.0 and ≤1.2 during the 10-day observation period [20]. The index is calculated as the mean of clinical scores (i.e., zero if normal, one if sick, two if very sick or paralyzed, and three if dead) per bird per observation (i.e., day).

### 3.6. Miscellaneous Assays to Detect Variant and Novel AIVs

Other than the above-mentioned laboratory tests that are used routinely depending on their need, there are tests used mainly for research purposes. Some of these tests are mentioned below.

#### 3.6.1. DNA Microarrays

DNA microarrays are based on the principle that complementary DNA strands bind with each other. Briefly, several thousand to millions of synthesized probes are pasted to a solid microchip. The pasted nucleotide sequences are complementary to segments of one or more target organism genome(s).

Mcloughlin (2011) has extensively reviewed DNA microarrays for pathogen detection and analysis [114]. ViroChip is one such microarray used to detect emergent influenza virus variants, such as the H1N1pdm09 [109]. For influenza virus detection, RNA from samples is extracted and converted to complementary DNA. The DNA is then amplified, fragmented, and labeled with a fluorescent dye. The labeled DNA is incubated on the microchip for several hours. During this time, if complementarity exists, the test DNA binds to probes. The unbound sequences in the sample are washed away. Subsequently, the surface of the microchip is checked for the presence of any bound sequences (i.e., targets). The fluorescence intensity is directly proportional to the number of probes complementary to the target DNA in the sample, leading to an estimate of virus quantity [114]. Photos of microarray DNA oligonucleotides of influenza viruses can be found in the following publication [115].

ViroChip offers several advantages in AIV diagnostics and surveillance. ViroChip is useful for known viruses and their variants [109]. Its major advantage is its capacity to detect several viruses simultaneously. Some limitations of ViroChip include its high cost and the need for specialized equipment and expertise, making it unsuitable for all clinical laboratories. Its usefulness is limited when applied to detecting novel viruses as it only detects viruses included in the chip’s probe library.

PhyloFlu is another DNA microarray for determining the phylogenetic origin of IAV gene segments and the genomic fingerprint of viral strains [116]. A DNA microarray-based multiplex assay of AIV subtypes H5, H7, H9, N1, and N2 has also been developed [117,118].

#### 3.6.2. Electron Microscopy

Electron microcopy (EM) is a classic technique that can be used for visualizing the presence of a viral agent in bodily fluids and tissues. However, it requires a sizable number of viruses present in the sample (~10^7^ virions/mL) [119]. It also requires advanced skills to prepare the sample. The sensitivity of EM can be enhanced by incorporating virus-specific antibodies into sample preparation to aggregate virus particles (i.e., immuno-EM). Besides virus concentration, sample selection should be based on the sites of abundant virus replication. EM can be beneficial for viruses that are difficult to isolate and can be used on both fluids (vesicle fluid, urine, feces) and tissue samples. Since EM is pathogen-independent, it is unbiased for the detection of unknown pathogens [120]. The use of EM for the direct visualization of influenza virus in samples has been reported [121].

Electron micrographs of negatively stained viral particles can be found in the following publications [122,123]. Nishida et al. (2019) used three-dimensional observation of the influenza virus surface by an ultra-high resolution scanning electron microscope [124].

#### 3.6.3. Mass Spectroscopy

Mass spectroscopy (MS) is an analytical technique that measures the mass-to-charge ratio (*m*/*z*) in ionized compounds [125]. Matrix-assisted laser desorption ionization–mass spectrometry (MALDI-MS) has been used for AIV identification [126]. Han et al. (2023) successfully applied MALDI-time-of-flight (TOF) MS to IAV subtyping [127]. Briefly, the sample is mixed with an energy-absorbent, organic compound called matrix. A laser beam ionizes the sample within the matrix. This generates singly protonated ions of various sizes from analytes within the sample. Acceleration of protonated ions under a fixed potential separates them from each other in a proportion relative to the *m*/*z*. Time of flight analyzers measure the charged analytes, which generate a peptide mass fingerprint (PMF). The information generated by the MALDI-TOF MS can be used to identify microbes, including AIV, in the following two ways: one possibility is matching the PMF of the unknown organism with that of a known organism in a database, and the other is to match the mass of the unknown organism’s protein biomarkers with that of a known organism’s biomarkers in a protein databank [125]. MALDI-TOF MS has also been employed in combination with antibody-magnetic nanoparticles to detect and rapidly screen IAV subtypes. For instance, the assay was sensitive enough to detect ~10^3^ EID_50_/_mL_ of LPAIV H5N2 from allantoic fluid within just one hour [128]. The significant improvement in the efficiency and speed of testing can be attributed to the combined use of aminosilane-coated iron oxide nanoparticles containing mAbs against HA protein on their surface, and subsequent detection of the LAPIV from lysate for direct MALDI-TOF MS without the tiresome elution step. An MS proteotyping approach has also been used to type, subtype, and identify lineages of human IAVs [129]. A recently developed liquid chromatography coupled with a tandem MS proteotyping method was able to successfully identify many pathogenic viruses, including IAV [130].

The MS suffers from poor sensitivity compared to PCR-based methods. Sample preparation for MS analysis can be complex and time-consuming, often involving steps like protein extraction, purification, and digestion, leading to a longer turnaround time for diagnosis. Interpreting MS data can also be challenging and may require specialized expertise. Complex algorithms and bioinformatics tools are often necessary to analyze the large datasets generated by MS experiments. MS instrumentation and reagents are expensive, potentially increasing the cost of diagnostics.

#### 3.6.4. Biosensors

Biosensors are tiny devices made up of a recognition component, a transducer, and an amplifier. The component detects the analyte and produces information, which the transducer transforms into usable signals such as sound, light, or electricity. The amplifier boosts these signals for further analysis. Biosensors are user-friendly, portable, and offer real-time analyses [60]. Digital temperature sensors for wireless avian-influenza monitoring in poultry farms have been developed [131,132]. The detailed information on the use of biosensor technologies for the detection of AIV is mentioned elsewhere [133].

## 4. Application of Diagnostic Assays for AIV Surveillance

Active and/or passive surveillance of poultry for AIV has been utilized by various countries, depending upon needs and resources. Testing methods also vary depending on the surveillance program. In passive surveillance, the infection status of a flock is suspected by the appearance of clinical signs resembling avian influenza. Upon noticing suspicious clinical signs, the producer can confer with a veterinarian and/or authority, who collects samples for laboratory testing for AIV, including rRT-PCR assays for AIV detection and then for H5 and H7 subtyping [134]. In the United States, establishing the US National Animal Health Laboratory Network (NAHLN) facilitates federal agencies and regional laboratories in testing samples originating from poultry mortalities for AIV (both suspect and outbreak). For active surveillance, a system similar to US NPIP can be designed and deployed to serologically survey participating poultry flocks using a commercial ELISA as the primary screening test. All ELISA-positive samples must be tested by an AGID test [134]. Alternatively, the AGID test can be used directly on poultry sera without first testing the samples with ELISA. An ELISA can also be used for active surveillance by testing individual birds with a competitive ELISA. The ELISA-positive samples can further be tested by HI test to confirm the subtype of AIV infecting the flock. The NPIP also supports the use of cloacal swabs from domestic ducks and poultry for AIV matrix gene rRT-PCR using a specific commercial kit for the NPIP notifiable avian influenza US H5/H7 Avian Influenza Clean and the US H5/H7 Avian Influenza Monitored Programs. The rRT-PCR procedure remains a screening test, and all positive findings need to be further tested as provided in 9 CFR 145.14(d) and 9 CFR 146.13(b) [134].

Generally, early detection of LPAIV infection presents a challenge, as clinical signs of avian influenza are lacking or not apparent. Early detection of LPAIV in commercial breeding flocks, laying hens, and turkey flocks can be achieved by periodic sampling for testing. Yet, such a sampling scheme may be difficult to achieve in broilers because of their short lifespan, although periodic testing of oropharyngeal swabs and sera from 5-week-old broilers could provide useful information on their disease status (David Halvorson, Department of Veterinary and Biomedical Sciences, College of Veterinary Medicine, University of Minnesota, Personal Communication, 2017). So-called “barrel surveillance” was employed with reasonable success for the 2002 H7N2 LPAI outbreak in Virginia, USA [135]. Target poultry species were commercial turkeys and broilers, including breeders of both species. “Barrel surveillance” involved collecting daily dead birds in barrels at the end of each premise’s driveway. The collected specimens were evaluated with ACIA, rRT-PCR, and VI. The authors claimed that the rRT-PCR could be used as the sole diagnostic test in the outbreaks of LPAIV infection [135]. However, in light of the effective use of serology by USDA and individual States for AIV surveillance, further evidence may be required to support the sole use of molecular testing for surveillance. Besides bird testing, environmental testing (water, droppings, air) has been utilized for AIV monitoring and surveillance purposes [136,137,138,139]. While environmental testing has been more common to detect AIV activity related to wild birds, the US poultry industry has adopted PCR testing of poultry facility’s environmental samples (e.g., slat floors, walls, cages, surfaces associated with egg processing, pits, and surfaces associated with manure handling, drinkers, sills, curtains, and frames) after the HPAI outbreak in 2015 to monitor the presence of AIV in the environment after USDA required cleaning and disinfecting premises or facilities [140]. Environmental testing is useful for detecting a pathogen in facilities or houses that could pose a risk to animals. Environmental testing is also a biosecurity measure that helps evaluate the cleaning and disinfection of animal facilities. In addition, environmental testing may help monitor an infectious agent’s presence in a premise during the acute phase of an outbreak of a disease. However, a recent study involving environmental sampling of H5N2 HPAIV (2014–2015) affected poultry facilities in the US Midwest suggested prolonged survival of AIV RNA without viable virus in the premises. This study observation suggests that molecular testing of poultry facility environmental samples alone may not be effective for surveillance, particularly after cleaning and disinfection but needs to be augmented with virus isolation or sentinel testing [141].

Bird market testing is another option for surveillance. In the US, live bird markets (LBMs), auction markets, and live bird dealers are surveyed at least once a month by the USDA Veterinary Services. LBM surveillance started in the mid-1980s and was scaled up in the 1990s. Additionally, all birds entering an LBM must be tested for AIV, and test records must be maintained for at least one year. While the state can test the birds at an LBM at any time, testing must be performed at least quarterly [142]. AIV surveillance data gathered from LBMs can be an early warning for domestic poultry producers. Rapid tests, such as lateral flow immunoassay, ELISA, PCR- or LAMP-based assays, and VI can be used to monitor LBMs for AIVs.

In resource-limited countries, various diagnostic assays can be utilized for AIV surveillance and monitoring. The choice of diagnostic assays depends on factors such as cost, availability of equipment, fitness for purpose, and trained personnel. For example, antigen-capturing immunoassays, which can detect specific viral proteins (i.e., antigens), are often preferred due to their simplicity and rapid results. These tests are especially useful in remote areas with limited laboratory infrastructure. The tests provide quick, on-site (pen-site) results, often within minutes. However, RT-PCR and virus isolation may be required for confirmation and characterization of an AIV actively circulating in poultry. Serology helps in identifying historic infections. By carefully selecting and utilizing appropriate diagnostic assays, resource-limited countries can also effectively monitor and control avian influenza outbreaks, protecting both human and animal health.

Machine learning (ML) is a branch of artificial intelligence applications that utilize statistical procedures to obtain novel insights into disease surveillance systems [143]. Walsh et al. (2019) developed an ML model based on matrix gene rRT-PCR, to maximize the probability of AIV isolation from the archived samples. The model can also be used to evaluate AIV surveillance designs, especially in resource-constrained laboratory settings [143]. 

A comparative summary of various diagnostic tests commonly used for AIV surveillance and monitoring is provided in Supplementary Table S1 [144,145]. 

## 5. Conclusions

Choosing the best diagnostic assays for a surveillance or monitoring program plays a pivotal part in determining its success. The assay’s choice should be evaluated based on the goal of testing, the time of testing during the disease course, the assay target, the sample matrix, assay sensitivity and specificity, and the advantages and disadvantages of the assay (Supplementary Table S1).

Rapid tests to detect viable viruses are currently lacking and are highly desired for the success of future surveillance and monitoring programs. The lack of standardization of various diagnostic assays is another challenge. Some AIV assays suffer from poor sensitivity and/or specificity. Moreover, diagnostic assays that can rapidly subtype multiple AIV subtypes are needed. Developing sensitive and specific assays with the ability to detect viable AIV and multiple AIV subtypes that can be deployed in the field could greatly support AIV surveillance and monitoring.

Here is the authors’ perspective on the surveillance and monitoring of AIV in migratory wild birds and poultry. Virus isolation will likely continue to be used as a standard method for AIV surveillance in migratory wild birds (MWBs) and poultry, yet it will remain critical for virus characterization. Antigen-capturing immunoassays with rapid turnaround time will continue to be used as a pen-side/on-site test for surveillance in clinically ill poultry. Detection and subtyping of AIVs directly from oropharyngeal-cloacal swabs by rRT-PCR will likely increase. Loop-mediated isothermal amplification-based assays will be commonly used for on-site testing in resource-limited countries. However, its use for AIV surveillance will depend on overcoming the technical limitations of the assay mentioned above. NASBA’s use for routine surveillance has been limited, but cost reduction may make this technology’s use more affordable and frequent. Serologic screening of poultry will likely continue. While the HI assay has been limited for surveillance, its use for routinely monitoring poultry flocks to check the development of protective AIV antibody titer after vaccination will likely continue. Additionally, HI assays employing mAbs against NP and HA viral proteins—resulting in simultaneous detection and subtyping of AIVs—will likely increase. The development and application of robotics in automating HI testing could be a major leap in increasing the use of this assay in routine surveillance. Sanger sequencing will continue to be routinely used as a reliable method for AIV characterization. However, recently emerged, high-throughput NGS technologies—with the ability to sequence all AIV segments in a single run—can replace Sanger sequencing. Minimizing cost, simplifying data analyses, and enhancing bioinformatics could make NGS technologies more applicable for the routine characterization of field viruses. Animal welfare concerns will require surveillance efforts to use non-invasive alternative sampling methods such as environmental sampling including feather sampling [139,142].

In summary, the continued AIV surveillance of MWB and domestic poultry using appropriate diagnostic assays and alternative sampling is warranted. Future surveillance efforts with improved tools must be expanded to understudied host species. A holistic understanding of AIV ecology can be obtained by surveying AIVs in the reservoir species, at their interface with other birds and animals, and the environment using a One Health approach.

## Supplementary Materials

The following supporting information can be downloaded at: https://www.mdpi.com/article/10.3390/v17020228/s1, Table S1: A comparison of laboratory assays commonly used for avian influenza virus surveillance and monitoring.
