# Supplementary material for: Diagnostic Assays for Avian Influenza Virus Surveillance and Monitoring in Poultry
Source: Viruses. 2025 Feb 6;17(2):228. doi: 10.3390/v17020228 (PMC11860460; doi:10.3390/v17020228)
Supplement: Supplementary file 1 [file viruses-17-00228-s001.zip › viruses-3263195-supplementary.pdf]

Table 1. A comparison of laboratory assays commonly used for avian influenza virus surveillance and monitoring.

Test	Target	Sample types	Applicable to			Advantages	Disadvantages
			GP	DW	WW		
rRT-PCR	Viral RNA (M or NP gene)	Oropharyngeal, cloacal or tracheal swabs, tissues	Yes	Yes	Yes	- Relatively very high sensitivity and specificity<br>- Rapid turnaround<br>- High throughput<br>- Quantification possibility	- Expensive<br>- Limited size of PCR product<br>- Potential cross-talk between different dyes<br>- False-negative results due to genetic mutation or assay inhibition<br>- False-positive results due to cross-contamination
VI	Live virus	Oropharyngeal, cloacal, or tracheal swabs, tissue homogenates, environmental samples	Yes	Yes	Yes	- Relatively very high sensitivity<br>- Producing virus isolates for characterization and vaccine production	- Relatively moderate specificity<br>- Labor intensive<br>- Requires appropriate sample collection and handling for virus viability<br>- Long turnaround (~ 2 weeks)<br>- Not easy to scale up<br>- Requires a good level of laboratory skills
ACIA	Viral nucleoprotein	Oropharyngeal, cloacal, or tracheal swabs	Yes	?	?	- Availability of commercial kits<br>- Relatively high specificity<br>- Inexpensive and easy to use<br>- Can be used on-site<br>- Rapid turnaround time	- Relatively low sensitivity
ELISA	Antibody to NP	Serum, plasma, egg yolk	Yes	Yes	Yes, but not validated[1]	- High throughput<br>- Automation capability<br>- Availability of commercial kits	- Relatively moderate sensitivity and specificity<br>- Need for an ELISA reader
AGID	NP and M protein	Virus isolates	Yes	NR[2], but have been used[3]	NR[2], but have been used[3]: poor sensitivity[3]	- Relatively very high specificity<br>- Broadly detects IAVs irrespective of the subtype<br>- Does not require specialized laboratory equipment	- Relatively moderate sensitivity<br>- Expensive reference antigen and antisera<br>- Laborious to prepare<br>- Longer turnaround time (~48 hours compared to ELISA & PCR (~2-3 hours)
	Antibody to NP and M protein	Serum, plasma, egg yolk	Yes	NR[4]	NR[4]	- Easy to conduct	
HI	HA protein	Virus isolates	Yes	Yes[3]	Yes	- Relatively very high sensitivity<br>- Inexpensive	- Relatively moderate to high specificity
	Antibody to specific HA subtype	Serum	Yes	?	No[5]	- Relatively easy to perform	
NI	NA protein	Virus isolates	Yes	?	?		- Relatively moderate sensitivity<br>- Relatively moderate to high specificity
	Antibody to specific NA subtype	Serum	Yes; but limited value for turkey sera[6]	?	?		- Relatively moderate specificity

Adapted and modified from Spackman et al. 2017 [24]. IAV detection (Active infection ~1-7 days post-infection); IAV antibody (Historic infection ~10-35 days post-infection). GP: Gallinaceous poultry, DW: Domestic waterfowl, WW: Wild waterfowl, rRT-PCR: real-time reverse transcription-polymerase chain reaction, VI: virus isolation, ACIA: antigen-

capture immunoassay, ELISA: enzyme-linked immunosorbent assay, AGID: agar gel immunodiffusion, NR: Not recommended, NP: nucleoprotein, HI: hemagglutination inhibition, NI: neuraminidase inhibition. 1:Brown et al. 2010 [144]; 2:Jenson, 2020 [84]; 3: Spackman, et al. 2009 [77]; 4: USDA, 2021[81]; 5:Lu et al. 1982 [145]; 6:Pedersen, 2014 [98].
